# Association of CASC18/miR-20a-3p/TGFB2 ceRNA axis with occult lymph node metastasis in tongue squamous cell carcinoma

**DOI:** 10.1186/s10020-021-00345-9

**Published:** 2021-08-06

**Authors:** Bo Zhou, Yue Zhou, Ying Liu, Hailin Zhang, Huangxing Mao, Mingjing Peng, Anji Xu, Zan Li, Hui Wang, Haolei Tan, Huayi Ren, Xiao Zhou, Ying Long

**Affiliations:** 1 Translational Medicine Centre, Hunan Cancer Hospital and the Affiliated Cancer Hospital of Xiangya School of Medicine, Central South University, 283 Tongzipo Road, Changsha, 410013 Hunan People’s Republic of China; 2 Department of Head and Neck Surgery, Hunan Cancer Hospital and the Affiliated Cancer Hospital of Xiangya School of Medicine, Central South University, Changsha, 410013 Hunan People’s Republic of China; 3 Hunan Provincial Clinical Research Centre for Oncoplastic Surgery, Hunan Cancer Hospital and the Affiliated Cancer Hospital of Xiangya School of Medicine, Central South University, Changsha, 410013 Hunan People’s Republic of China; 4 Key Laboratory of Translational Radiation Oncology, Hunan Cancer Hospital and the Affiliated Cancer Hospital of Xiangya School of Medicine, Central South University, Changsha, 410013 Hunan People’s Republic of China; 5 Hunan Traditional Chinese Medical College, Zhuzhou, 412012 Hunan People’s Republic of China

**Keywords:** CASC18, TGFB2, ceRNA, Occult lymph node metastasis, Tongue squamous cell carcinoma

## Abstract

**Background:**

Tongue squamous cell carcinoma (TSCC) ranks as the most prevalent malignancy in the oral cavity. TSCC patients with occult lymph node metastasis (OLNM) are thought to be at risk of worse outcome. However, regulatory mechanisms underlying OLNM remain less investigated.

**Methods:**

In the present study, CASC18/miR-20a-3p/TGFB2 axis was identified and evaluated by bioinformatic and qRT-PCR analyses. Effects of CASC18 knockdown on cell migration and invasion were determined by wound healing and transwell assays. Western blot, ELISA, RNA pulldown and luciferase reporter assays were performed for mechanism verification.

**Results:**

CASC18 was identified up-regulating in TSCC tumours, and especially in those from patients with OLNM. Importantly, we found higher CASC18 expression was positively correlated with the presence of OLNM and worse outcome of TSCC patients. Furthermore, we demonstrated that CASC18 knockdown repressed cell migration and invasion through inhibiting epithelial-mesenchymal transition, which could be partly rescued by miR-20a-3p inhibitor. Regarding the molecular mechanism, we further confirmed that CASC18 functioned as a ceRNA to sponge miR-20a-3p to enhanceTGFB2 expression and secretion.

**Conclusion:**

In conclusion, we have reported a novel CASC18/miR-20a-3p/TGFB2 ceRNA axis in OLNM of TSCC. Our findings will contribute to a deeper understanding of the molecular mechanism of OLNM in TSCC, and facilitate the development of diagnostic methods for assisting treatment decision-making.

**Supplementary Information:**

The online version contains supplementary material available at 10.1186/s10020-021-00345-9.

## Introduction

Tongue squamous cell carcinoma (TSCC) is the most prevalent malignancy in the oral cavity, with an increasing incidence in the past decades (Bray et al. 2018; Miller et al. 2019; Gulland and Gulland 2016). The presence of lymph node metastasis is one of the most accurate predictors of prognosis for TSCC patients. With advances in detection and treatment, TSCC outcomes have improved in recent years (Jansen et al. 2018). However, approximately 20–40 % of TSCC patients with a clinical N0 (cN0) neck are subsequently diagnosed with micro-metastasis in the lymph nodes after careful pathological examination (Ganly et al. 2012; Bilde et al. 2008). These patients harboring occult lymph node metastasis (OLNM) would miss the opportunities of therapeutic neck dissection unless their clinical N stage is accurately assessed prior to treatment. Thus, dissecting the molecular mechanisms involved in OLNM is of great clinical importance.

Recent high-throughput studies are contributing to an improved understanding of the molecular mechanism concerning various phenotypes, such as carcinogenesis (Sun et al. 2018), metastasis (Tian et al. 2017), and chemoresistance (Dai et al. 2019). These cancer-associated phenotypes are at least partly controlled by differentially expressed genes (DEGs), including long non-coding RNA (lncRNA), microRNA (miRNA), and protein-coding gene (PCG). Recently, these three types of molecules can be linked together according to the competing endogenous RNA (ceRNA) regulatory hypothesis (Salmena et al. 2011). Some lncRNAs can act as miRNA sponges to regulate the expression of PCG through competitively binding to shared miRNAs. Although increasing evidence has been well documented that ceRNA network plays pivotal roles in many cancers (Qi et al. 2015), even in TSCC, current knowledge for roles of ceRNA regulation in OLNM of TSCC is not enough.

In the present study, we first identified the ceRNA axes mediated by aberrantly expressed lncRNAs in TSCC with OLNM via mining the sub-dataset of TCGA-HNSC project. Functional enrichment analysis was performed to reveal the potential roles of this OLNM-related lncRNA. Through bioinformatic prediction and in vitro experiments, we further confirmed that CASC18 functions as a ceRNA to regulate cell migration and invasion via miR-20a-3p/TGFB2 axis. Our results provide novel insights into the ceRNA regulation mechanism and reveal potential roles of CASC18 in OLNM of TSCC.

## Materials and methods

### Tissues and cell line

34 FFPE samples of TSCC were collected with the consent of patients, and the experiments were approved by the ethics committee of Hunan Cancer Hospital (KYJJ-2020-222), Changsha, China. TSCC cell line CAL27 was purchased from Shanghai Genechem Co., Ltd, and cultured in DMEM with 10 % FBS (Gibco, Gaithersburg, MD,) as routine. For RNAi, siRNAs (si-CASC18#1: ACCTAGTCATACATCCTAA; si-CASC18#2: CCTTATCCCTGGATGGAAA) were transfected into CAL27 cells with Lipofectamine 2000 (Invitrogen, Carlsbad, CA) for 48 h.

### Acquisition and DEG screening of RNA-seq data

The gene expression data and clinical information of TCGA-HNSC subset were downloaded from The Cancer Genome Atlas (TCGA; cancergenome.nih.gov), and data of TSCC patients were filtered. Subsequently, the DESeq2 (version 1.22) package (https://bioconductor.org/packages/DESeq2/) was used to screen for TSCC- and OLNM-related DEGs with the cut-off values of |log2FC|≥1 (where FC is fold change)and adjusted p < 0.05 (Love et al. 2014). The ggplot2 (version 3.3, cran.r-project.org/package = ggplot2) and ggpubr (version 0.2.3, https://rpkgs.datanovia.com/ggpubr) packages were used to display the results as volcano plots (Maag and Maag 2018; Ito et al. 2013). Then, the intersection of the DEGs was calculated, and the result was visualized as Venn diagrams using the VennDiagram package (https://CRAN.R-project.org/package=VennDiagram) in R (version 3.4.3; www.r-project.org) (Chen et al. 2011) . To identify the correlation between differentially expressed lncRNAs and PCGs, the Pearson correlation coefficient (PCC) value was subsequently calculated for each pair of lncRNA-PCG. Pairs with |PCC| > 0.45 (p < 0.05) and corrected p-value < 0.05 were considered as statistically significant correlation.

### Functional enrichment analysis

The WEB-based GEne SeT AnaLysis Toolkit (WebGestalt, version 2019, http://www.webgestalt.org/) (Liao et al. 2019) was applied to map candidate PCGs, correlated with differentially expressed lncRNAs, onto their associated biological annotation, with Gene Ontology (GO; www.geneontology.org) (Gene Ontology Consortium 2017; Ashburner et al. 2000). All significantly enriched terms, with adjusted p < 0.05, were visualized by using the ggcorrplot (version 0.1. 3, CRAN.R-project.org/package = ggcorrplot) package.

### Fluorescence in situ hybridization (FISH)

FISH was executed to determine the subcellular location of CASC18 in CAL27 cells. Fluorogenic probes targeting CASC18, 18 S and U6 were designed and synthesized by RiboBio (Guangzhou, China). Hybridization was carried out overnight according to the manufacturer’s instructions. All fluorescent images were captured with the same exposure setting and illumination.

### qRT-PCR

For Formalin-Fixed and Paraffin-Embedded (FFPE) samples, the total RNA was extracted from at least four 10-µm FFPE scrolls using the RNeasy FFPE Kits (QIAGEN, Germantown, MD), and then reverse transcribed to cDNA using PrimeScriptTM RT-PCR Kit (Takara, Dalian, China) in accordance with the manuals. The expression level of CASC18 was detected, and samples were divided into low and high expression groups by the median value of this lncRNA. Subsequently, comparison of VIM (vimentin) and CDH1 (E-cadherin) expression between two groups were performed. For cells, nuclear and cytoplasmic RNA were isolated using the PARIS™ Kit (Ambion), and the total RNA was extracted by using Trizol reagent (Invitrogen, Carlsbad, CA). For miRNA quantitation, reverse transcriptions were performed using the PrimeScript RT Reagent Kit (Takara, Dalian, China) with specific stem-loop primers, designed and synthesized by RiboBio (Guangzhou, China). qRT-PCR was performed using SYBR^®^ Premix DimerEraser™ (Takara, Dalian, China) in Roche LightCycler 480 II Real-Time PCR system (Roche, Basel, Switzerland). The threshold cycle value (Ct) of each product was determined and normalized against that of the internal control of β-actin (for lncRNA/PCG) or U6 (for miRNA), and the differences were compared by t-test using SPSS 23.0, with p < 0.05 considered as statistically significant. Primer pairs for gene expression quantitation were as follows: CASC18-F: 5′-TGTATAGTCTAGCCAAGTCC-3′, CASC18-R: 5′-ATTTCAGCCATCTTCAGTCCC-3′, β-actin-F: 5′-ACCCTGAAGTACCCCATCGAG-3′, β-actin-R: 5′-AGCACAGCCTGGATAGCAAC-3′, VIM-F: 5′-CGAGGAGAGCAGGATTTCTC-3′, VIM-R: 5′-GGTATCAACCAGAGGGAGTGA-3′, CDH1-F: 5′-TGCCCAGAAAATGAAAAAGG-3′, CDH1-R: 5′-TGCCCAGAAAATGAAAAAGG-3′.

### ELISA

The concentration of TGF-β2 in CAL27 culture supernatants was measured by using ELISA kit (Cloud-Clone corp., Wuhan, China), according to the manufacturer’s instructions.

### Luciferase reporter assay

The fragments of CASC18-wt (wildtype) and CASC18-mut (miR-30a-3p binding site mutation) were ligated into pcDNA3.1 vector to form two plasmids. Vectors for Luciferase reporter assay were generated based on the pmirGLO Dual-Luciferase miRNA Target Expression Vector (Promega, Madison, WI). Then, pmirGLO-TGFB2 3′-UTR-wt (wildtype) and pmirGLO-TGFB2 3′-UTR-mut (miR-20a-3p binding site mutation) were constructed, and the plasmids was co-transfected with miR-20a-3p mimics, negative control, pcDNA3.1-CASC18-wt, and pcDNA3.1-CASC18-mut into CAL27 cells with Lipofectamine 2000 according to the guidelines, respectively. The relative luciferase activity was measured with the Dual-Luciferase Reporter Assay System (Promega, Madison, WI).

### RNA pulldown

The biotinylated DNA probe complementary to CASC18 and was synthesized (GenePharm, Shanghai, China), with a random probe as the control. M-280 streptavidin magnetic beads (Sigma-Aldrich, St. Louis, MO) were coated by incubating with the probes, respectively. The cell lysates of CAL27 transfected with si-CASC18#2 or si-NC were prepared after 48 h. Lysates were then incubated with the probe-coated beads at 4 °C overnight, and molecules interacted with CASC18 were captured after washing. The bound RNAs were subsequently purified using TRIzol and the miR-20-3p abundance was finally measured by qRT-PCR.

### Wound healing assay

As cells at 80 % confluence in a six-well culture dish, the monolayer was scraped with a sterile 200 µl pipette tip. The cells were cultured in a culture medium with 3 % FBS and 1 % penicillin/streptomycin (TransGen, Beijing, China). Images were captured at 0, 24, and 48 h after the scratch was made. Image-Pro Plus v6.0 image analysis software was used to analyse the cell wound healing rate as described in our previous study (Dong et al. 2019).

### Transwell invasion assay

The transwell invasion assay was performed using the transwell (Corning, NY, USA) and matrigel (BD Biosciences, San Jose, CA) as described previously (Justus et al. 2014), with slight modifications. Cells were added into the upper chamber. After 36 h of incubation at 37 °C in 5 % CO_2_, cells invading through the matrigel and membrane to the lower surface were fixed and stained with Crystal violet for 10 min. The number of invaded cells were counted in 6 randomly selected visual fields under the microscope.

### Western blot

The harvested cells were lysed in RIPE buffer at 4 ℃ for 30 min and centrifuged at 15,000×*g* for 15 min for the protein sediment. Equal amount of proteins was loaded to SDS-polyacrylamide gel electrophoresis (SDS–PAGE) for separation, and then transferred to PVDF membranes (Millipore, Billerica, MA) according to the standard protocol. Subsequently, the membrane was incubated with primary antibodies (ProteinTech Group Inc., Chicago, IL) against TGF-β2 (1:2000 dilution), E-cadherin (1:1,000 dilution), N-cadherin (1:1000 dilution), Vimentin (1:1500 dilution) and β-actin (FN; 1:2000 dilution) overnight at 4 °C. After incubating with the corresponding secondary antibody for 1 h at room temperature, the immunoblotting signals were measure with enhanced chemiluminescence (ECL) kit (Pierce, Rockford, IL).

### Statistical analysis

Data were presented as mean ± SD from at least three separate experiments, with a p-value < 0.05 considered statistically significant. The Student t test, Wilcox test, log-rank test, or Fisher exact test was used for comparisons between groups. The Kaplan–Meier method was used to estimate overall survival.

## Results

### Identification of DEGs associated with OLNM in TSCC

**Fig. 1:**
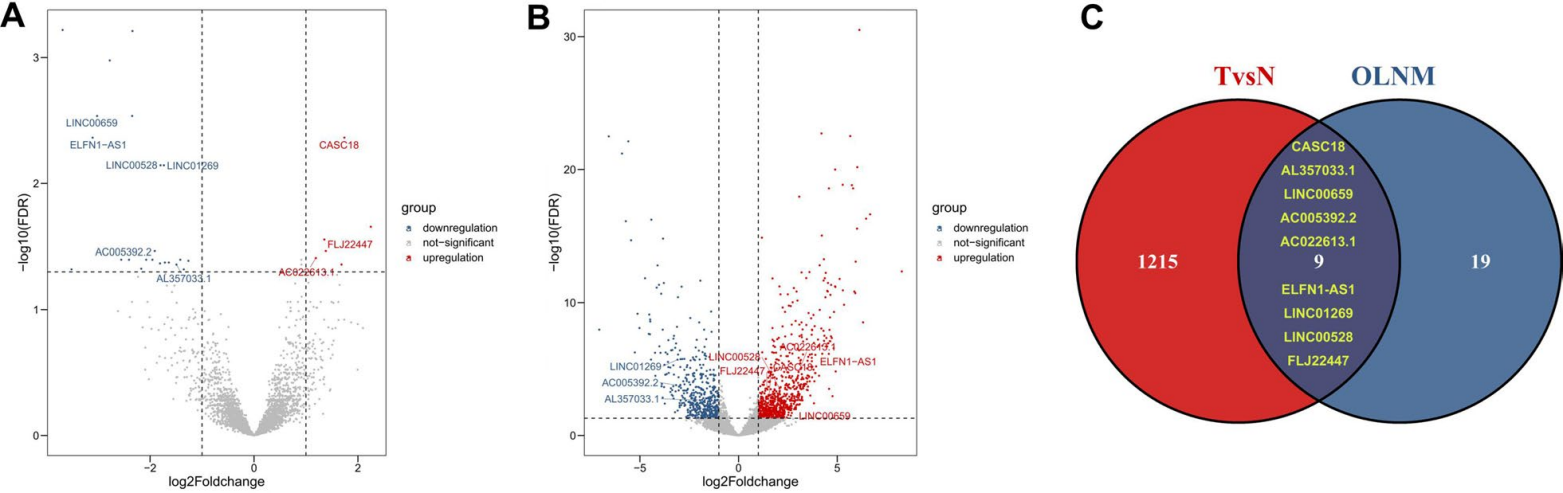
Identification of lncRNAs differentially expressed in tumors derived from TSCC patients with OLNM. Volcano plots exhibit DEGs **A** in TSCC tumors as compared with the normal tissues, and in **B** tumors derived from TSCC patients with or without OLNM, with the symbols of differentially expressed lncRNA indicated. Red color meant up-regulated genes, and the blue color meant down-regulated genes. **C** Schematic illustration exhibiting overlap of differentially expressed lncRNAs. Red represents differentially expressed lncRNAs related to TSCC, blue represents differentially expressed lncRNAs related to OLNM, and the overlapping area represents the commonly changed lncRNAs

A total of 53 cN0M0 TSCC patients were selected from the TCGA-HNSC cohort through filtration of TNM stage and anatomic sites. To understand expression differences between TSCC patients with and without OLNM, we carried out differential gene expression analysis and identified 28 differentially expressed lncRNAs (Fig. 1A). Among them, 6 genes were up-regulated and 22 genes were down-regulated (Additional file 1: Table S1). Then, expression data of 13 pairs of TSCC tissues and corresponding adjacent non-cancerous tongue tissues were obtained from TCGA-HNSC cohort. Comparing the TSCC tissues with the normal tongue tissues, a total of 1224 DEGs were identified, comprising 746 up-regulated lncRNAs and 478 down-regulated lncRNAs (Fig. 1B, Additional file 1: Table S2). Subsequently, when the DEGs were investigated for overlap, a total of 9 consistently aberrant genes were identified (Fig. 1C).

### Exploration of potential ceRNA axes by bioinformatics analysis

**Fig. 2:**
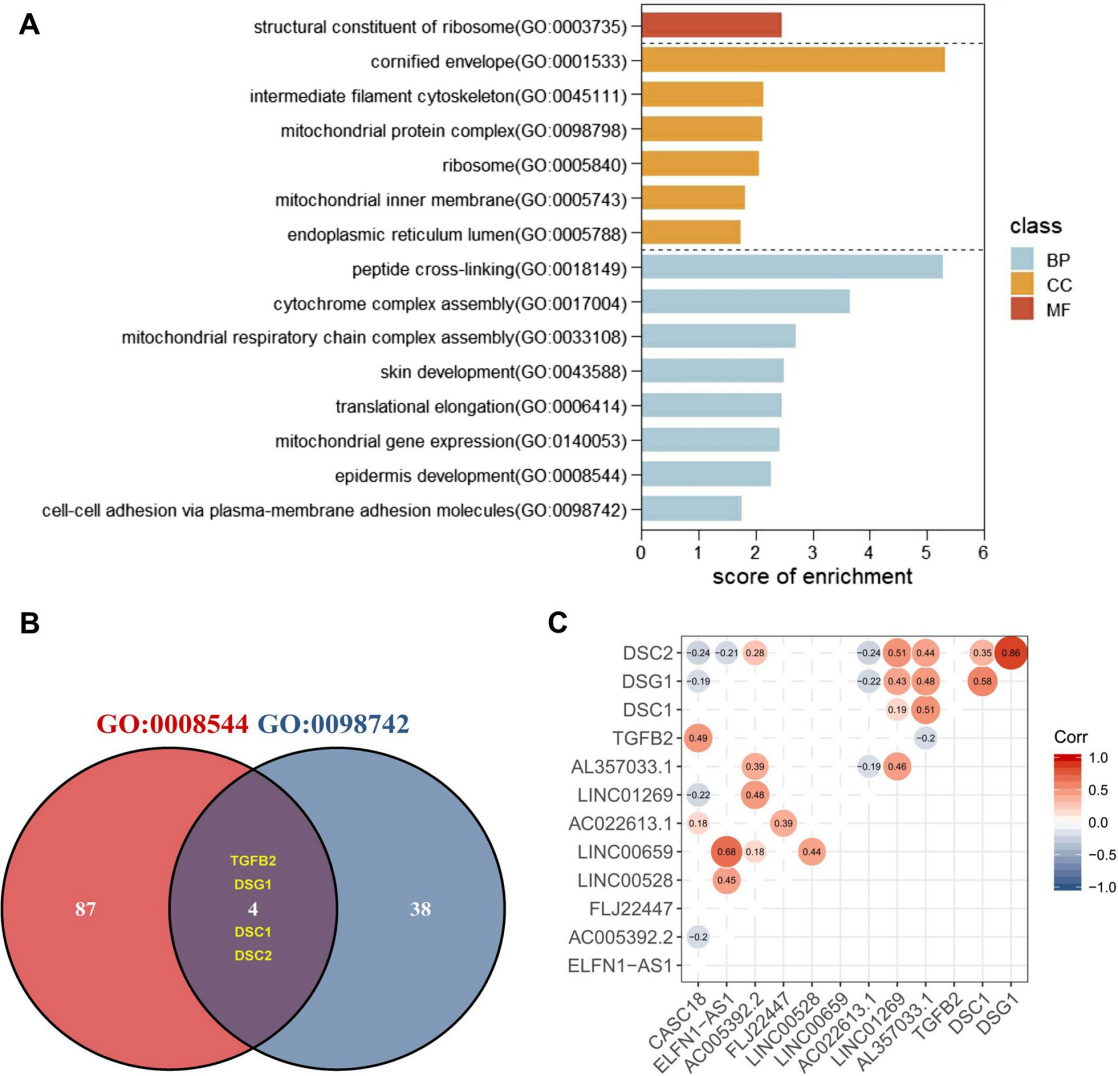
Functional enrichment and correlation analyses of candidate lncRNA-related PCGs **A** Enriched GO terms of candidate lncRNA-related PCGs were visualized as a bar graph. BP meant biological process, CC meant cellular component, and MF meant molecular function. **B** Schematic illustration exhibiting overlap of enriched PCGs. Red represents genes enriched in GO: 0008544, and blue represents genes enriched in GO: 0098742. **C** Corrplot presentation of correlation between 9 lncRNAs and 4 PCGs

**Fig. 3:**
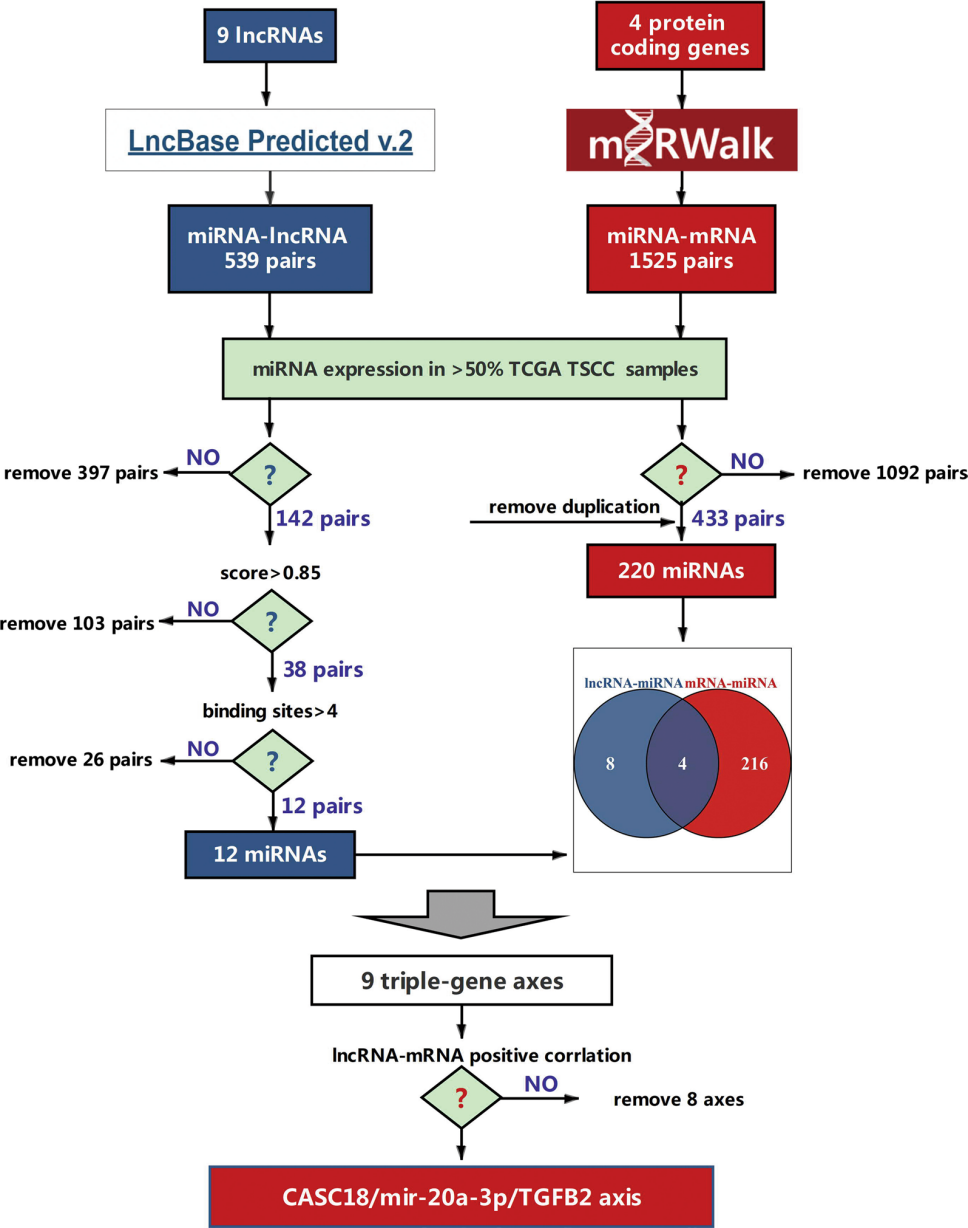
Flowchart for ceRNA axes screening

The correlation between each lncRNA and PCG expression was evaluated by the Pearson correlation coefficient (PCC) analysis in the TCGA-TSCC subset. A total of 1758 PCGs were found correlated with at least one of 9 common lncRNAs (PCC > 0.45, p < 0.05). Subsequently, these 1758 PCGs were subjected to GO enrichment analysis. As shown in Fig. 2A, they were enriched significantly into 15 diverse GO terms, being categorized into three functional groups: Biological process (BP), cellular component (CC) and molecular function (MF). Among these terms, “cell-cell adhesion via plasma-membrane adhesion molecules” was the term related to cancer metastasis, and abnormal expression of genes in “epidermis development” may partly contribute to tumorigenesis of TSCC. Then, overlapping genes, TGFB2, DSG1, DSC1 and DSC2, were identified between mentioned two terms by the intersecting analysis (Fig. 2B). The correlation of 9 lncRNAs to 4 PCGs was visualized in Fig. 2C. With a multi-step filtration strategy (Fig. 3), we then investigated the potential regulatory axes between each lncRNAs and PCGs. A total of 539 miRNA-lncRNA pairs were predicted by lncBase computational methods. After removal of miRNAs (1) expressed in less than 50 % TCGA-TSCC samples, (2) with interaction score < 0.85, (3) with < 5 binding sites in the target lncRNA, 12 candidate miRNAs were selected. By using miRWalk computational methods, 1525 miRNA-mRNA pairs were obtained. A list of 220 miRNAs was collected after deleting miRNAs expressed in less than 50 % TCGA-TSCC samples. Overlapping the lncRNA-related miRNA and mRNA-related miRNA, we selected 4 candidate miRNAs and formed 9 triple gene axes. Interestingly, only the CASC18/miR-20a-3p/TGFB2 axis met lncRNA-mRNA positive correlation, and thus was selected for further validation.

### Expression and characterization of CASC18 in TSCC

**Fig. 4:**
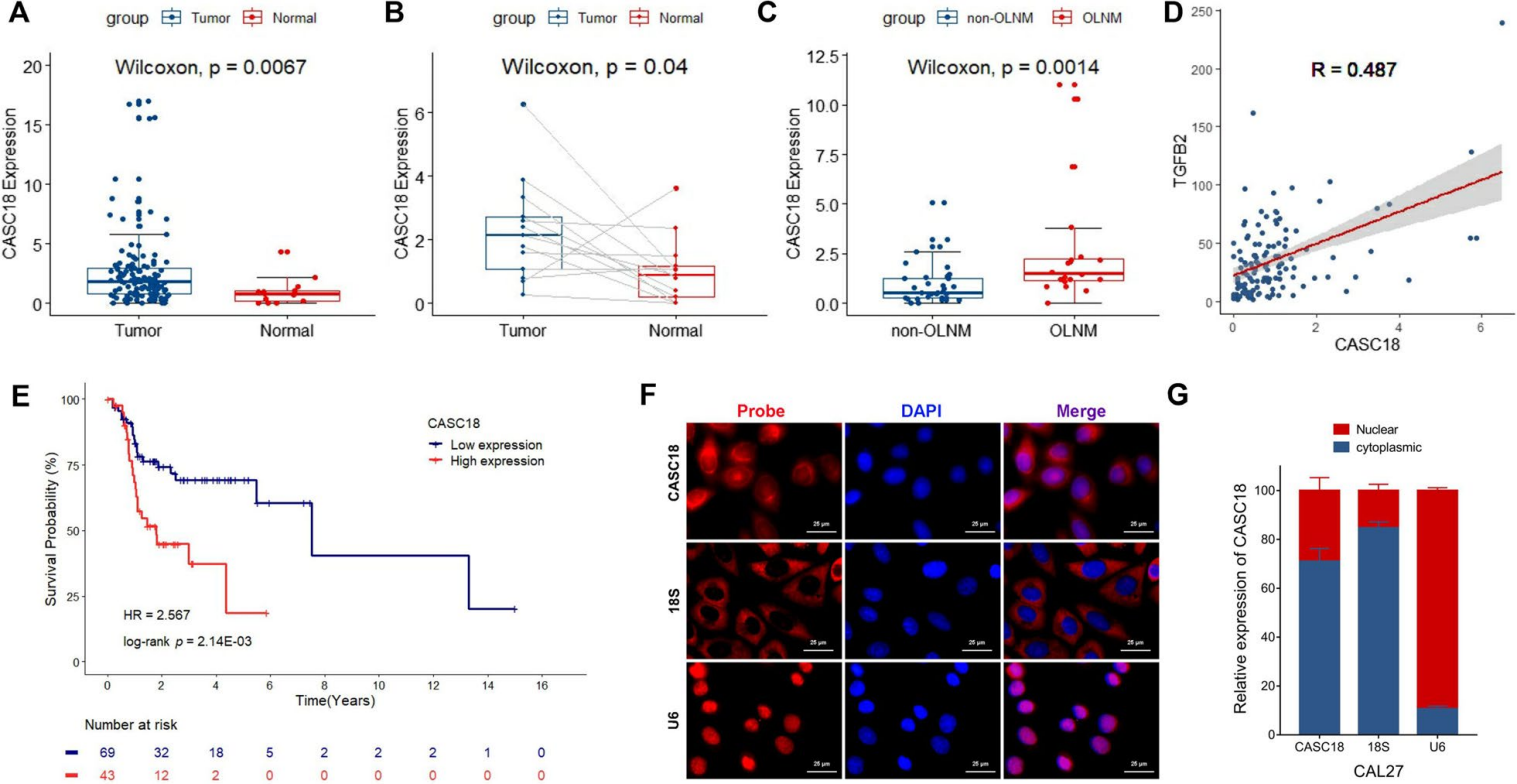
The expression pattern and clinical characteristics of CASC18 in TCGA-TSCC cohort and CAL27 CASC18 is highly expressed in tumors as compared with **A** the normal tissues, **B** matched adjacent tissue in TCGA-TSCC cohort. CASC18 is **C** highly expressed in tumors derived from patients with OLNM as compared with those without OLNM, **D** positively correlated with TGFB2 expression, and **E** poor prognosis predictor. **F** The subcellular distribution of CASC18 was assessed by FISH staining in CAL27 cells, with the reference of 18 S and U6. **G** The abundance of CASC18 in nuclear and cytoplasmic RNA was determined and normalized against that of the internal control of 18 S and U6. Data are presented as mean ± SD, with a p-value < 0.05 considered statistically significant

In TCGA-TSCC cohort, the significantly lower level of CASC18 expression was detected in normal tongue tissues when compared with TSCC (p = 0.007) but also corresponding tumor tissues (p = 0.040), respectively (Fig. 4A, B). Moreover, Fig. 4C showed that CASC18 obviously enhanced in tumor samples of TSCC patients with OLNM (p = 0.001). Results of PCC analysis (Fig. 4D) showed markedly positive correlation between CASC18 and TGFB2 (PCC = 0.487, p < 0.01). What is more important, increased CASC18 expression is associated with worse prognosis of patients in TCGA-TSCC cohort (Fig. 4E). To further investigate the subcellular localization of this lncRNA, fluorescence in situ hybridization (FISH) and quantitative real-time PCR (qRT-PCR) assays were performed. The results demonstrated that CASC18 was predominantly localized in the cytoplasm (Fig. 4F, G), implying that CASC18 can act as a sponge of miRNA and function as a ceRNA in cytoplasm. Then, we further examined CASC18 expression in an independent cohort of 34 TSCC FFPE samples, and determined the correlation between their CASC18 expression and clinicopathologic characteristics. As shown in Table 1, high CASC18 expression was positively associated with TSCC tumors characterized by the Pathologic N stage (p = 0.006) as well as OLNM (p = 0.006).

**Table 1:** Relationship between CASC18 expression and clinicopathologic characteristics of TSCC patients

Clinical information	CASC18 (n = 34)		p-value
	Low expression	High expression	
Age at diagnosis (year)			1.000
< 51	7 (41.2 %)	10 (58.8 %)	
≥ 52	10 (58.8 %)	7 (41.2 %)	
Sex			0.493
Female	4 (23.5 %)	4 (23.5 %)	
Male	13 (76.5 %)	13 (76.5 %)	
Smoking			0.303
No	11 (64.7 %)	7 (41.2 %)	
Yes	6 (35.3 %)	10 (58.8 %)	
Clinical T stage			0.224
T1	7 (41.2 %)	3 (17.6 %)	
T2	10 ( 58.8 %)	13 (76.5 %)	
T3	0 (0.0 %)	1 (5.9 %)	
Pathologic T stage			0.338
T1	4 (23.5 %)	1 (5.9 %)	
T2	11 (64.7 %)	13 (76.5 %)	
T3	2 (11.8 %)	3 (17.6 %)	
Pathologic N stage			0.006
N1	14 (82.4 %)	5 (29.4 %)	
N2	1 (5.9 %)	8 (47.1 %)	
N3	2 (11.8 %)	4 (23.5 %)	
OLNM			0.006
No	14 (82.4 %)	5 (29.4 %)	
Yes	3 (17.6 %)	12 (70.6 %)	

### Knockdown of CASC18 inhibited cell migration and invasion in vitro

**Fig. 5:**
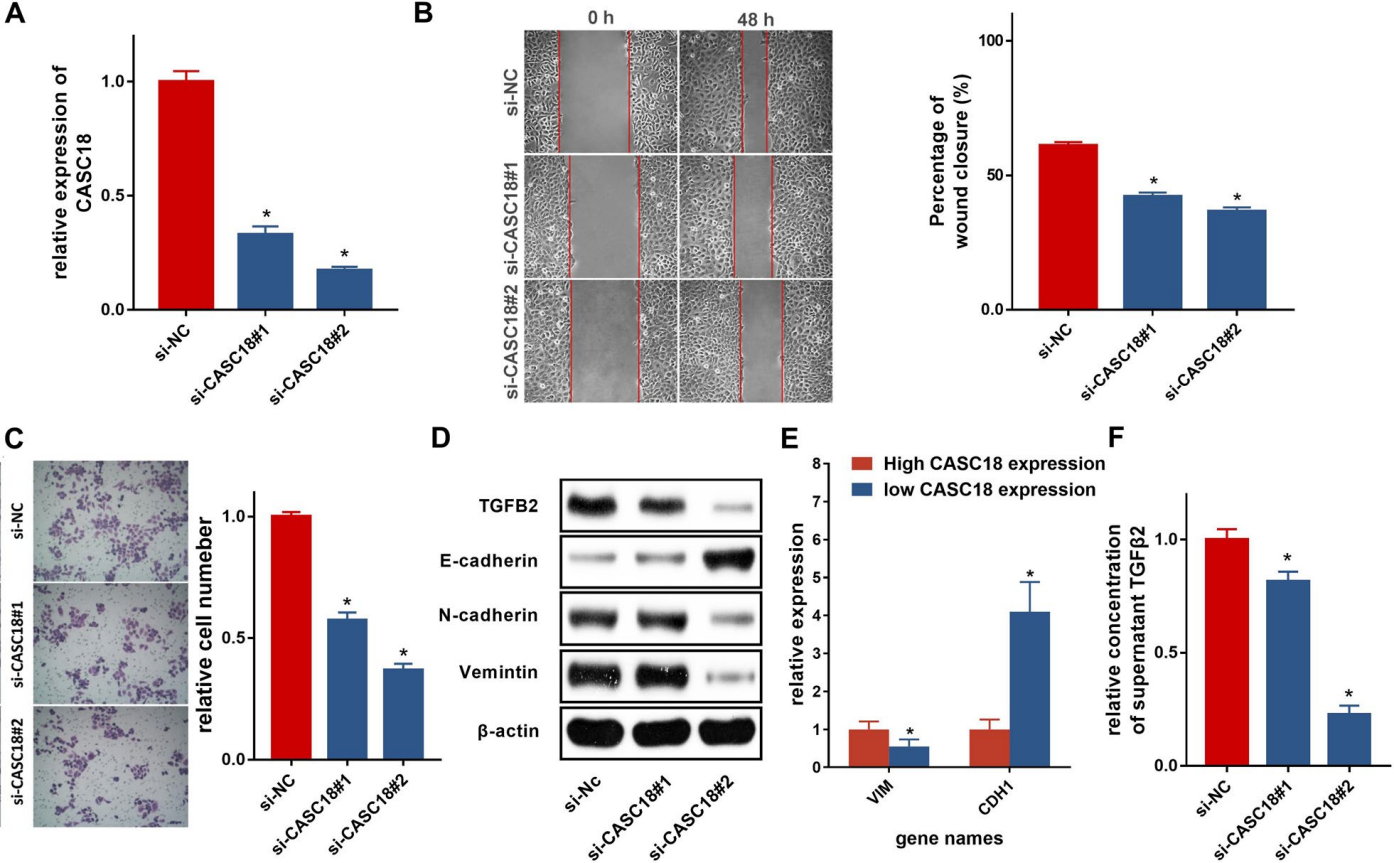
Silencing CASC18 inhibits CAL27 cell migration and invasion through repressing TGFB2-mediated EMT **A** The silencing of CASC18 was conducted with the transfection of si-CASC18#1 and si-CASC18#2, as compared with si-NC. Representative photos of **B** wound healing and **C** assays indicate the role of CASC18 RNAi in cell migration and invasion. The right histograms denote the percentage of wound closure and the relative number of invaded cells, respectively. The impact of CASC18 knockdown on the **D** expressions of TGFB2, E-cadherin, N-cadherin, Vimentin by western blotting analysis with the reference of β-actin. **E** The mRNA levels of VIM and CDH1 genes were detected by qRT-PCR in two groups of clinical samples carrying high or low CASC18 expression. **F** The impact of CASC18 knockdown on the secretion of TGFB2 by ELISA. Data are presented as the mean ± SD. *p < 0.05

To investigate the biological function of CASC18, we interfered its expression by transient transfecting of siRNA in CAL27 cells. Obviously, si-CASC18#2 displayed the most significant suppression on silencing CASC18 (Fig. 5A). Wound healing assay showed that CASC18 knockdown repressed cell migration in CAL27 cells, and si-CASC18#2 displayed a greater inhibitory effect (Fig. 5B). Likewise, the transwell assay also indicated the suppressive effect of CASC18 downregulation on cell invasion (Fig. 5C). However, MTT assay did not show a significant effect of CASC18 expression on cell proliferation (data not shown). Considering the association of epithelial-mesenchymal transition (EMT) with cell mobility and invasion, as well as the key role of TGF-β pathway in EMT, we further detected the expression level of TGF-β2 and EMT-related markers by western blot. Our result revealed that expression of TGF-β2, as well as N-cadherin and Vimentin, which are mesenchymal markers, was decreased; whereas expression of E-cadherin, an epithelial marker, was alternatively increased after CASC18 RNAi (Fig. 5D). Additionally, results of qRT-PCR indicated that VIM was highly expressed in FFPE samples carrying high CASC18 expression, while CDH1 was highly expressed in those carrying low CASC18 expression (Fig. 5E). Besides, we found that CASC18 knockdown significantly decreased TGF-β2 secretion in CAL27 culture supernatants (Fig. 5F). These results suggested the correlation among CASC18, TGFB2 and EMT in promotion of CAL27 cell migration and invasion.

### CASC18 acts as a miR-20a-3p sponge to prompt CAL27 cell migration and invasion via regulating TGFB2 expression

**Fig. 6:**
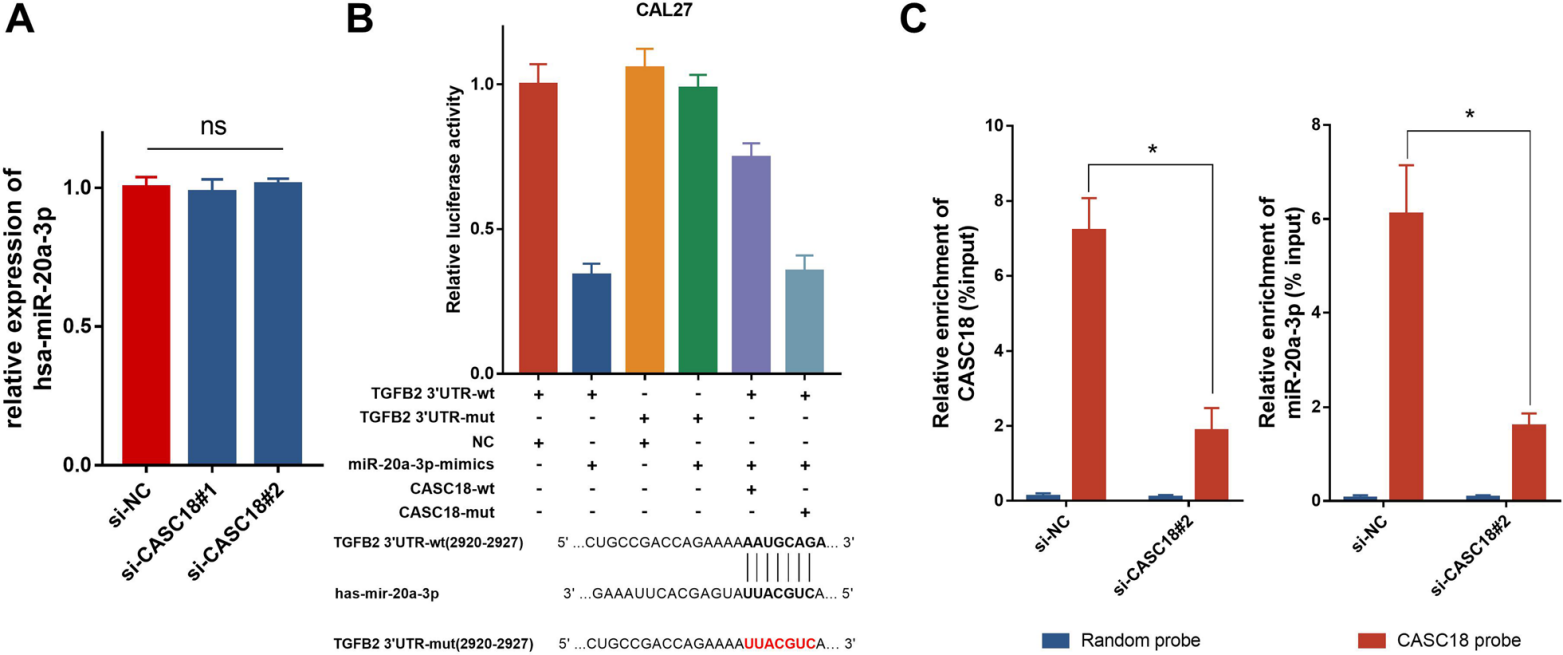
CASC18 positively regulates TGFB2 by acting as the miR-20a-3p sponge **A** The impact of CASC18 knockdown on the expressions of miR-20a-3p in CAL27 by qRT-PCR. **B** Luciferase activity in CAL27 cells co-transfected with siRNAs and miRNA inhibitors. **C** RNA pulldown assay was executed in CAL27 cells, followed by qRT-PCR to detect the enrichment of CASC18 and miR-20a-3p. Data are presented as the mean ± SD. *p < 0.05

**Fig. 7:**
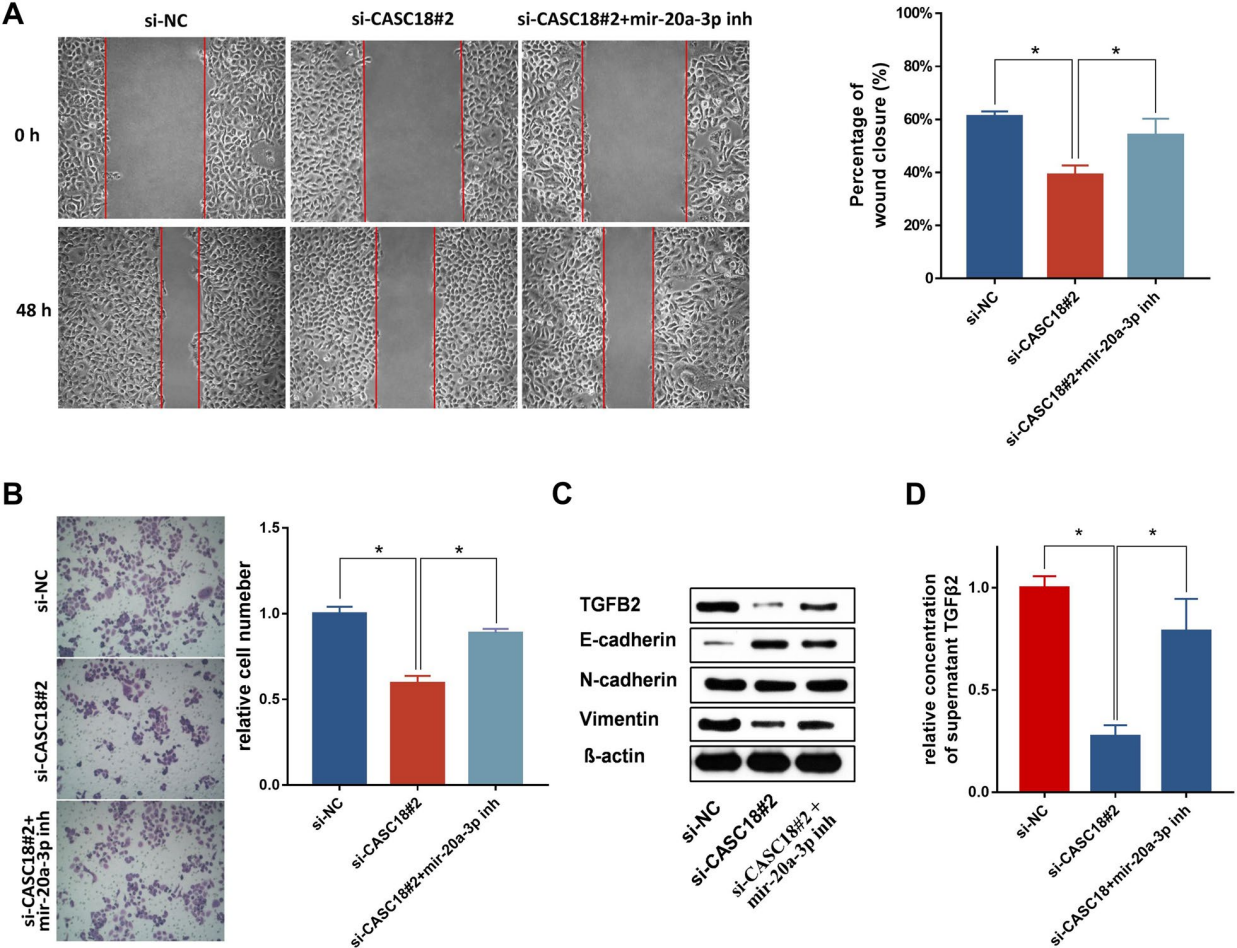
Inhibitor against miR-20a-3p attenuates the repression effects of CASC18 knockdown on cell migration and invasion The effect of miR-20a-3p inhibitor on **A** cell migration and **B** invasion of CASC18-silencing CAL27 was evaluated by wound healing and transwell assays, respectively. The right histograms denote the percentage of wound closure and the relative number of invaded cells, respectively. The impact of miR-20a-3p inhibitor on **C** the expression of TGFB2 and EMT-related biomarkers and **D** secretion of TGFB2 in CASC18-silencing CAL27 by wester blot and ELISA, respectively. miR-20a-3p inh meant miR-20a-3p inhibitor. Data are presented as the mean ± SD. *p < 0.05

**Fig. 8:**
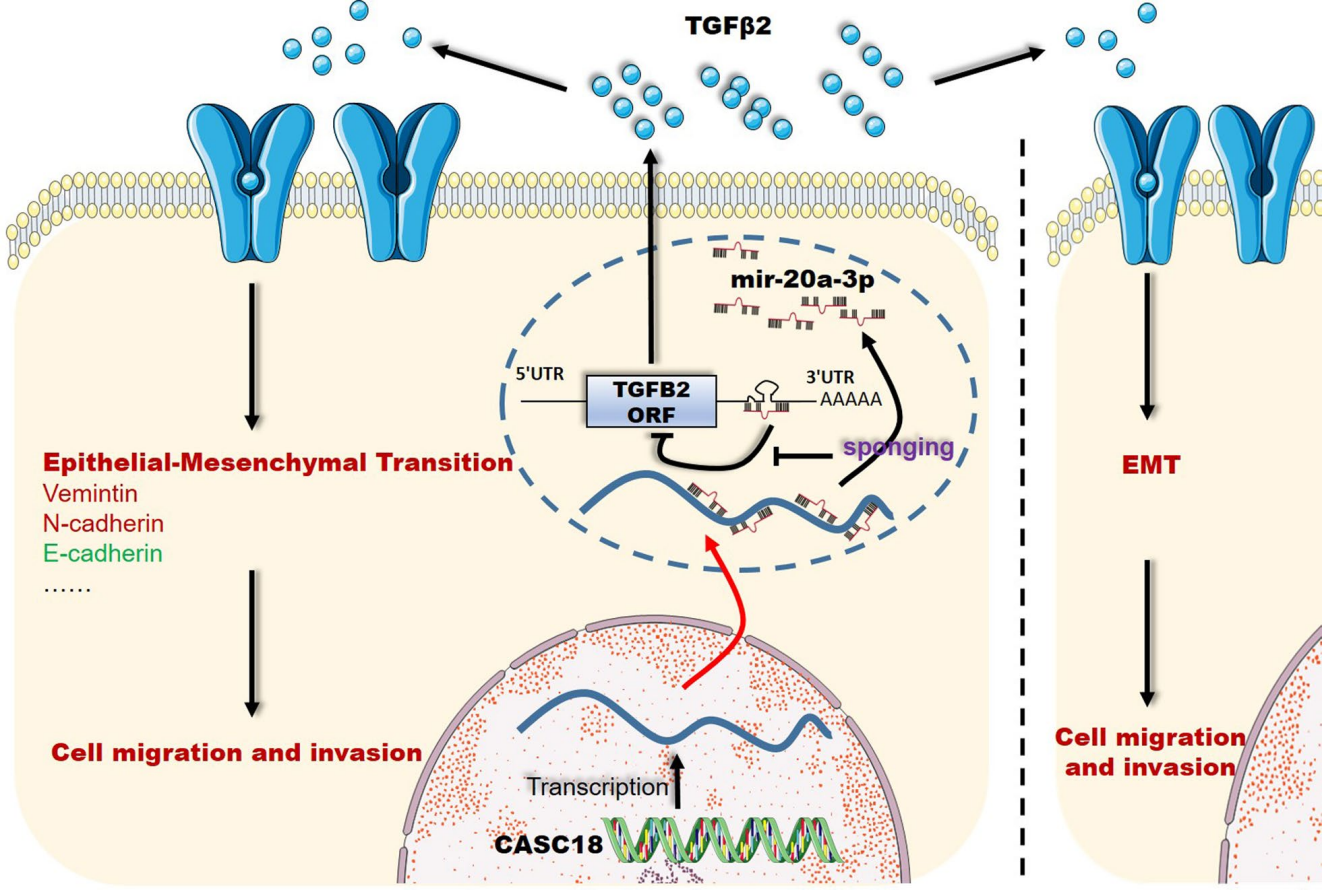
Schematic diagram of CASC18/miR-20a-3p/TGFB2 ceRNA axis involved in cell migration and invasion of TSCC CASC18 stimulates TGFB2 expression and secretion via sponging miR-20a-3p, and thus promotes TSCC cell migration and invasion through TGFB2-mediated EMT in an autocrine and/or paracrine manner

To compare levels of miR-20a-3p in CAL27 cells after CASC18 knockdown, we transfected CAL27 cell with siRNAs against CASC18 and si-NC. The expression of miR-20a-3p unchanged with the decrease of CASC18 (Fig. 6 A), indicating that CASC18 did not regulate miR-20a-3p expression. We then constructed luciferase plasmids containing a TGFB2 wildtype 3′UTR (TGFB2 3′UTR-wt) or a mutated TGFB2 3′UTR (TGFB2 3′UTR-mut), whose miR-20a-3p binding site mutated. Luciferase assay showed that miR-20a-3p inhibitor significantly enhanced luciferase activity of TGFB2 3′UTR-wt, but it presented less effect on the TGFB2 3′UTR-mut (Fig. 6B). Moreover, CASC18 knockdown could also decrease the luciferase activity of TGFB2 3′UTR-wt, and this change can be partially eliminated by treating with the miR-20a-3p inhibitor. What is more important, miR-20a-3p inhibitor and CASC18 knockdown had no influence on the luciferase activity of TGFB2 3′UTR-mut, indicating regulation in a miR-20a-3p binding-site dependent manner. To next determine the binding property between miR-20a-3p and CASC18, RNA-pull down assay was performed. The biotinylated probe was firstly used to pulldown CASC18 in CAL27. (Fig. 6C, left panel). Subsequently, the enrichment of miR-20a-3p, which was captured by CASC18, was measured in CAL27. However, silencing of CASC18 significantly decreased the abundance of miR-20a-3p (Fig. 6C, right panel), indicating the binding interaction between miR-20a-3p and CASC18. The above results suggest that CASC18 elevates TGFB2 expression and secretion via sponging miR-20a-3p in TSCC cells. To further validate the role of CASC18/mir-20a-3p/TGFB2 axis in CAL27 cell migration and invasion, we performed the rescue experiments. As expected, exogenous miR-20a-3p inhibition significantly deprived the suppression role of cell mobility by CASC18 downregulation in CAL27, when compared with CASC18 RNAi alone (Fig. 7A, B). Moreover, results of western blot and Enzyme-linked immunosorbent assay (ELISA) revealed that expression and secretion of TGF-β2 were alternatively increased after miR-20a-3p inhibition in CASC18 knockdown CAL27 (Fig. 7C, D). Taken together, our results suggest that CASC18 stimulates EMT via relieving miR-20a-3p-mediated TGFB2 repression, and thus plays an ultimate role in regulating TSCC cell migration and invasion (Fig. 8).

## Discussion

For TSCC patients harbouring OLNM, prophylactic neck dissection was not routinely performed, attributing to the misclassification of a cN0 neck. Consequently, most of these patients suffered from the disease recurrence and metastases with a worse outcome. However, the molecular mechanisms involved are not clearly defined. In the present study, we found CASC18 expression was associated with the status of OLNM in TSCC cohorts, and acted as a ceRNA to modulate cell migration and invasion via miR-20a-3p/TGFB2 axis.

In the last decade, considerable studies have identified that lncRNAs played critical roles in carcinogenesis and metastasis (Lv et al. 2021; Yao et al. 2019; Poursheikhani et al. 2021). To data, the ceRNA hypothesis sparked new areas of lncRNA research on gene expression regulation, but some controversy still surrounded it (Tay et al. 2011; Smillie et al. 2018). The prediction of ceRNA axes, which is wholly dependent on the web tool for identifying shared MRE identification, is an overwhelming limitation of studies assessing ceRNA regulation(Thomson et al. 2016). This strategy is common in bioinformatics studies without experimental approach (Liu et al. 2020; Tang et al. 2020), and high false positive rate of their results is inevitable. Additionally, the cytoplasm abundance of miRNA is critical for its efficacy, and some lncRNA is expressed much higher for miRNAs with lower expression (Di Leva et al. 2013). Accordingly, we screened out the OLNM-related ceRNA axes using a multi-step filtration that takes into account the existence and number of MREs in lncRNA, positive correlation between lncRNA and associated-PCG, expression level of miRNA in TSCC samples, and computational interaction score, to discriminate signal from noise. Together with the intersection of PCGs enriched in both the terms of “cell-cell adhesion via plasma-membrane adhesion molecules” and “epidermis development”, only the CASC18/miR-20a-3p/TGFB2 axis, who fulfilled all the condition, was selected for further validation.

Although CASC18 named as Cancer Susceptibility 18, this gene was previously reported involved in neural cell differentiation and oocyte growth (Bouckenheimer et al. 2018; Mehravar et al. 2017), instead of cancer. To verify the function of the lncRNA in OLNM of TSCC, we first investigated the expression pattern and prognosis relationship of CASC18 in TCGA-TSCC subset. We found CASC18 is a prognosis factor up-regulated in tumour samples and especially in those of TSCC patients with OLNM, partly supporting the roles of CASC18 in lymph node metastases. Several models, including orthotopic tumour model (Eun et al. 2020), subcutaneous tumour xenograft model (Knudsen et al. 2021), tumour cell tail vein injection for metastasis model (Zhao et al. 2021), were extensively applied for cancer research. Regrettably, appropriate animal model for OLNM is still unavailable, and thus the association of CASC18 expression with OLNM was not verified in animal model in the present study. However, we found high CASC18 expression was positively correlated with the presence of OLNM in an independent TSCC cohort, which provides a clinical evidence for the association between CASC18 expression and OLNM in TSCC.

According to the predicted ceRNA axis, it has been easy to come away with the idea that CASC18 was involved in the regulation of EMT, due to the representative role of TGF-β signals in this process (Hao et al. 2019). EMT defines dynamic changes in cellular organization from epithelial to mesenchymal phenotypes, which is commonly considered to facilitate cell migration and invasion (Yang et al. 2020), particularly in cancers (Brabletz et al. 2018). Our in vitro experiments indicated that CASC18 influenced TGFB2 expression and secretion, and affected CAL27 cell migration and invasion. Consistent with previous studies (Thieme 2020; Jiang et al. 2021), EMT was inhibited with the attenuation of TGFB2 expression in CASC8-knockdown CAL27 cells. These data suggested that CASC18 regulated cell mobility via TGFB2-mediated EMT in TSCC cells. In order to further detect the underlying mechanism, we then investigated the subcellular localization of CASC18. As expected, this lncRNA was mainly distributed in the cytoplasm of CAL27 cells, in accordance with the basic requirement of ceRNA regulation (Salmena et al. 2011). To note, target-directed miRNA decay (TDMD) mechanism is another regulation mode analogous to ceRNA in some way, emphasizing the degradation of miRNA mediated by its binding target and ubiquitin ligase ZSWIM8 (Han et al. 2020; Shi et al. 2020; Sheu-Gruttadauria et al. 2019). To exclude the possibility of TDMD, we investigated miR-20a-3p expression after CASC18 knockdown, and our results indicated the stable expression of the miRNA in CAL27. Accordingly, we further verified the promising ceRNA regulation of CASC18/miR-20a-3p/TGFB2 axis. We confirmed that TGFB2 was a direct target of miR-20a-3p, and could be decreased by CASC18 knockdown in a miR-20a-3p-binding dependent manner by using luciferase reporter assays. Results of CASC18 pulldown assays further showed the direct binding of CASC18 and miR-20a-3p. Furthermore, we found that CASC18 knockdown suppressed the TGFB2 protein levels and secretion, but these effects were partly reversed by miR-20a-3p inhibitor treatment in CAL27 cells. Similarly, miR-20a-3p inhibitor treatment partly rescued the suppression effect of CASC18 knockdown on CAL27 cell migration and invasion. Taken together, our findings indicate that CASC18 functions as miR-20a-3p sponge to regulate cell migration and invasion via stimulating TGFB2-mediated EMT in CAL27.

## Conclusions

In conclusion, we have uncovered a novel CASC18/miR-20a-3p/TGFB2 axis and elaborated its involvements in OLNM of TSCC patients. Our study provided pioneering insights and fundamental clues for future thorough understanding of the molecular mechanism of TSCC OLNM, which might facilitate the development of improved diagnostic methods for treatment decision-making.

## Supplementary Information

**Additional file 1**: Lists of differentially expressed genes in this work.

## Data Availability

The datasets analysed during the current study are available in TCGA (cancergenome.nih.gov) repository. TCGA belongs to the public database and the patients involved in the database have obtained ethical approval. TCGA allows users to download relevant data for free for research and publish relevant articles.

## References

[CR1] Ashburner M (2000). Gene ontology: tool for the unification of biology. The Gene Ontology Consortium. Nat Genet.

[CR2] Bilde A (2008). Need for intensive histopathologic analysis to determine lymph node metastases when using sentinel node biopsy in oral cancer. Laryngoscope.

[CR3] Bouckenheimer J (2018). Differential long non-coding RNA expression profiles in human oocytes and cumulus cells. Sci Rep.

[CR4] Brabletz T, Kalluri R, Angela Nieto M, Weinberg RA (2018). EMT in cancer. Nat Rev Cancer.

[CR5] Bray F (2018). Global cancer statistics 2018: GLOBOCAN estimates of incidence and mortality worldwide for 36 cancers in 185 countries. CA Cancer J Clin.

[CR6] Chen H, Boutros PC (2011). VennDiagram: a package for the generation of highly-customizable Venn and Euler diagrams in R. BMC Bioinform.

[CR7] Dai P (2019). Screening candidate microRNA-mRNA network for predicting the response to chemoresistance in osteosarcoma by bioinformatics analysis. J Cell Biochem.

[CR8] Di Leva G (2013). Estrogen mediated-activation of miR-191/425 cluster modulates tumorigenicity of breast cancer cells depending on estrogen receptor status. PLoS Genet.

[CR9] Dong S (2019). HOXD-AS1 promotes the epithelial to mesenchymal transition of ovarian cancer cells by regulating miR-186-5p and PIK3R3. J Exp Clin Cancer Res.

[CR10] Eun Y-G, Yoon YJ, Won KY, Lee YC (2020). Circulating tumor DNA in saliva in an orthotopic head and neck cancer mouse model. Anticancer Res.

[CR11] Ganly I, Patel S, Shah J (2012). Early stage squamous cell cancer of the oral tongue–clinicopathologic features affecting outcome. Cancer.

[CR12] Gene Ontology Consortium. Expansion of the gene ontology knowledgebase and resources. Nucleic Acids Res 2017;45: D331-D338. 10.1093/nar/gkw1108 PMC5210579 27899567

[CR13] Gulland A (2016). Oral cancer rates rise by two thirds. BMJ.

[CR14] Han J, et al. A ubiquitin ligase mediates target-directed microRNA decay independently of tailing and trimming. Science 2020;370. 10.1126/science.abc9546 PMC8177725 33184234

[CR15] Hao Y, Baker D, Ten Dijke P (2019). TGF-beta-mediated epithelial-mesenchymal transition and cancer metastasis. Int J Mol Sci.

[CR16] Ito K, Murphy D (2013). Application of ggplot2 to pharmacometric graphics. CPT: Pharmacomet Syst Pharmacol.

[CR17] Jansen L (2018). Differences in incidence and survival of oral cavity and pharyngeal cancers between Germany and the United States depend on the HPV-association of the cancer site. Oral Oncol.

[CR18] Jiang XM (2021). TGFbeta2-mediated epithelial-mesenchymal transition and NF-kappaB pathway activation contribute to osimertinib resistance. Acta Pharmacol Sin.

[CR19] Justus CR, Leffler N, Ruiz-Echevarria M, Yang LV. In vitro cell migration and invasion assays. J Visualized Exp: JoVE 2014;51046. 10.3791/51046 PMC4186330 24962652

[CR20] Knudsen ES (2021). Targeting dual signalling pathways in concert with immune checkpoints for the treatment of pancreatic cancer. Gut.

[CR21] Liao Y, Wang J, Jaehnig EJ, Shi Z, Zhang B (2019). WebGestalt 2019: gene set analysis toolkit with revamped UIs and APIs. Nucleic Acids Res.

[CR22] Liu H, et al. The ceRNA network has potential prognostic value in clear cell renal cell carcinoma: a study based on TCGA database. Biomed Res Int. 2020;4830847. 10.1155/2020/4830847 PMC7335400 32685491

[CR23] Love MI, Huber W, Anders S (2014). Moderated estimation of fold change and dispersion for RNA-seq data with DESeq2. Genome Biol.

[CR24] Lv E, Sheng J, Yu C, Rao D, Huang W (2021). LncRNA influence sequential steps of hepatocellular carcinoma metastasis. Biomed Pharmacother.

[CR25] Maag JLV (2018). gganatogram: an R package for modular visualisation of anatograms and tissues based on ggplot2. F1000Research.

[CR26] Mehravar M (2017). Introduction of novel splice variants for CASC18 gene and its relation to the neural differentiation. Gene.

[CR27] Miller KD (2019). Cancer treatment and survivorship statistics, 2019. CA Cancer J Clin.

[CR28] Poursheikhani A, Abbaszadegan MR, Kerachian MA (2021). Mechanisms of long non-coding RNA function in colorectal cancer tumorigenesis. Asia Pac J Clin Oncol.

[CR29] Qi X (2015). ceRNA in cancer: possible functions and clinical implications. J Med Genet.

[CR30] Salmena L, Poliseno L, Tay Y, Kats L, Pandolfi PP (2011). A ceRNA hypothesis: the Rosetta stone of a hidden RNA language?. Cell.

[CR31] Sheu-Gruttadauria J (2019). Structural basis for target-directed MicroRNA degradation. Mol Cell.

[CR32] Shi CY, et al. The ZSWIM8 ubiquitin ligase mediates target-directed microRNA degradation. Science 2020;370. 10.1126/science.abc9359 PMC8356967 33184237

[CR33] Smillie CL, Sirey T, Ponting CP (2018). Complexities of post-transcriptional regulation and the modeling of ceRNA crosstalk. Crit Rev Biochem Mol Biol.

[CR34] Sun W, Qiu Z, Huang W, Cao M (2018). Gene expression profiles and proteinprotein interaction networks during tongue carcinogenesis in the tumor microenvironment. Mol Med Rep.

[CR35] Tang H, Wang Z, Shao Q, Wang Y, Yang Q (2020). Comprehensive analysis of competing endogenous RNA (ceRNA) network based on RNAs differentially expressed in lung adenocarcinoma using The Cancer Genome Atlas (TCGA) database. Med Sci Monit.

[CR36] Tay Y (2011). Coding-independent regulation of the tumor suppressor PTEN by competing endogenous mRNAs. Cell.

[CR37] Thieme R (2020). TGFB1 and TGFB2 Mediated Epithelial-Mesenchymal Transition in Esophageal Adenocarcinoma Cells. Oncology Research Treatment.

[CR38] Thomson DW, Dinger ME (2016). Endogenous microRNA sponges: evidence and controversy. Nat Rev Genet.

[CR39] Tian F, Zhao J, Fan X, Kang Z (2017). Weighted gene co-expression network analysis in identification of metastasis-related genes of lung squamous cell carcinoma based on the Cancer Genome Atlas database. J Thorac Dis.

[CR40] Yang J (2020). Guidelines and definitions for research on epithelial-mesenchymal transition. Nat Rev Mol Cell Biol.

[CR41] Yao R-W, Wang Y, Chen L-L (2019). Cellular functions of long noncoding RNAs. Nat Cell Biol.

[CR42] Zhao B, et al. (2021) A novel oncotherapy strategy, direct thrombin inhibitors suppress progression, dissemination and spontaneous metastasis in non-small cell lung cancer. Br J Pharmacol. 10.1111/bph.15384 33481255

